# Supplementary material for: Evaluation of an Ozone Chamber as a Routine Method to Decontaminate Firefighters’ PPE
Source: Int J Environ Res Public Health. 2021 Oct 9;18(20):10587. doi: 10.3390/ijerph182010587 (PMC8536115; doi:10.3390/ijerph182010587)
Supplement: Supplementary file 1 [file ijerph-18-10587-s001.zip › ijerph-1400687-supplementary.pdf]

International Journal of
Environmental Research
and Public Health

MDPI

Evaluation of an Ozone Chamber as a Routine Method to Decontaminate Firefighters' Gears

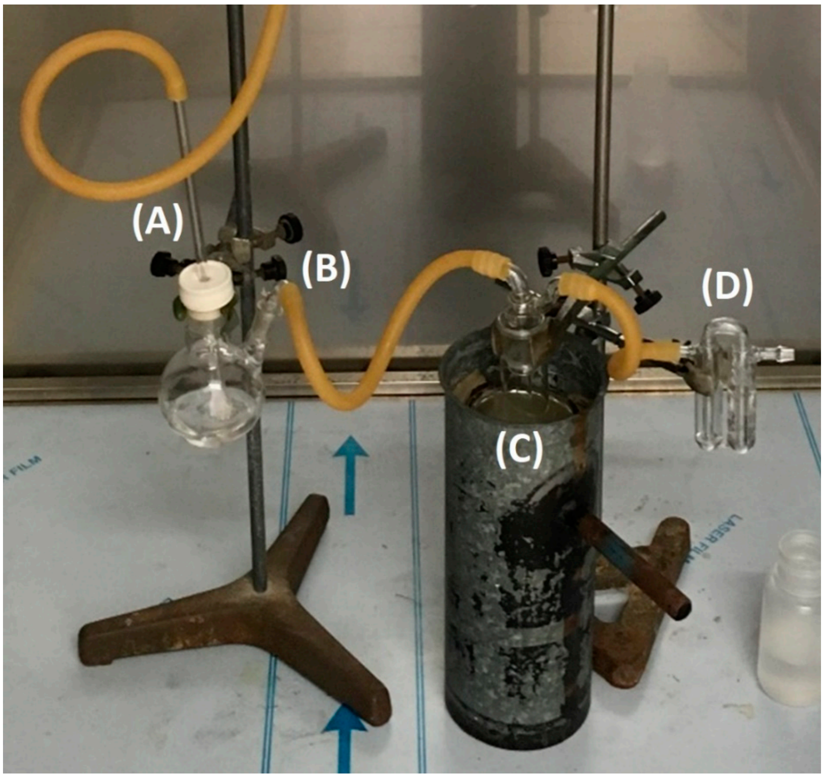

Figure S1. Setup used in the homogeneous experiments. (A) ozone input; (B) gas output; (C) cold trap system; (D) non-return gas trap system.

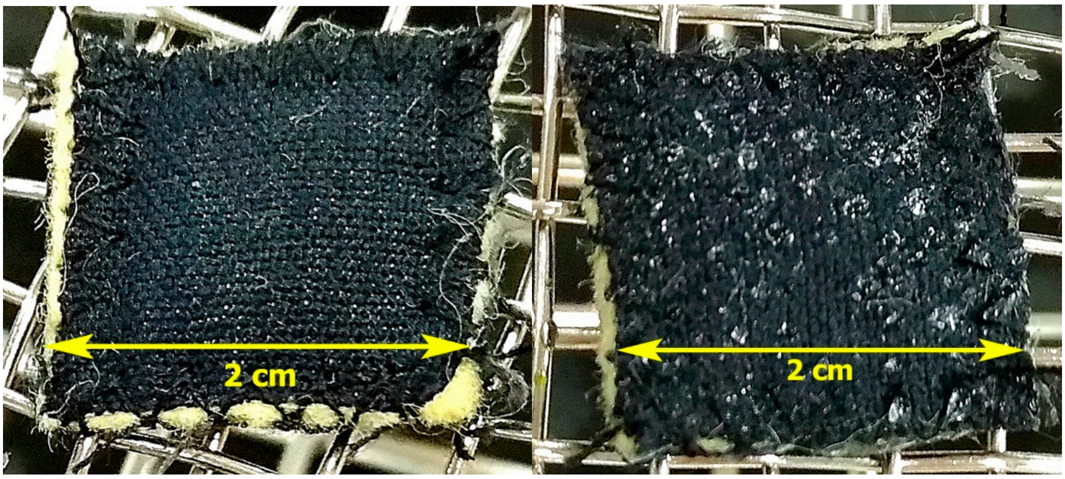

Figure S2. x 2 cm fragments of the firefighters' personal protective clothes (PPC) applied in the heterogeneous experiments (at dry and moistened conditions).Immediately before the ozonation, half of the fragments (15 of each compound) were sprayed with distilled water using an ordinary glass bottle spray (4 sprays on the inner surface and 4 on the outer one), to evaluate the influence of moisture on the ozonolysis reaction.

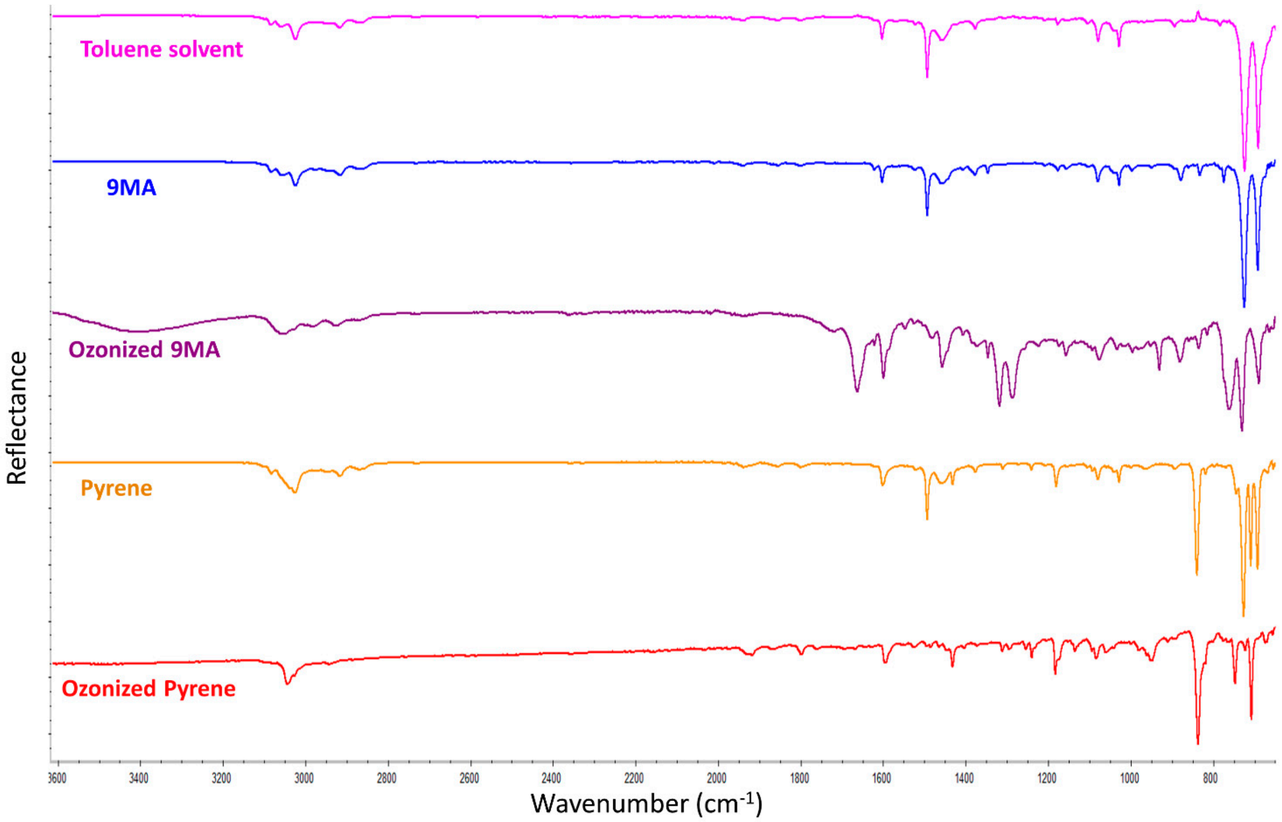

Figure S3. FTIR spectra of the 9-methylantracene (9MA) and pyrene standards in toluene solution, and after 60 min of ozone treatment in homogeneous experiments. The spectrum of the toluene solvent is also displayed. The FTIR spectra are vertically offset for clarity.

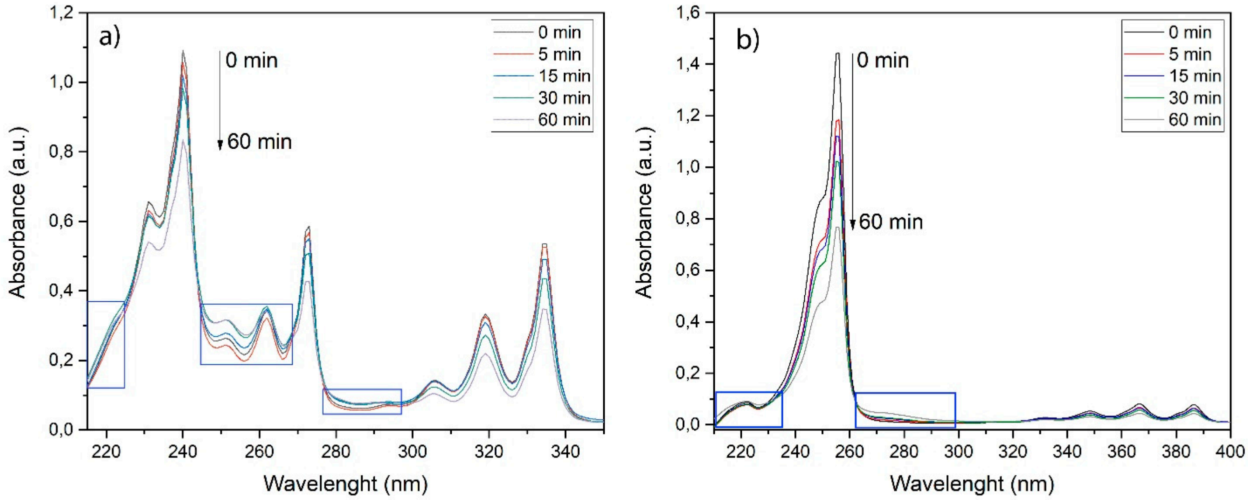

Figure S4. UV-Vis absorption spectra of the samples extracted from the PPE fragments with (a) pyrene and (b) 9-methylanthracene under moistened conditions after different ozonation treatments. Similar results than those obtained for the dried conditions.

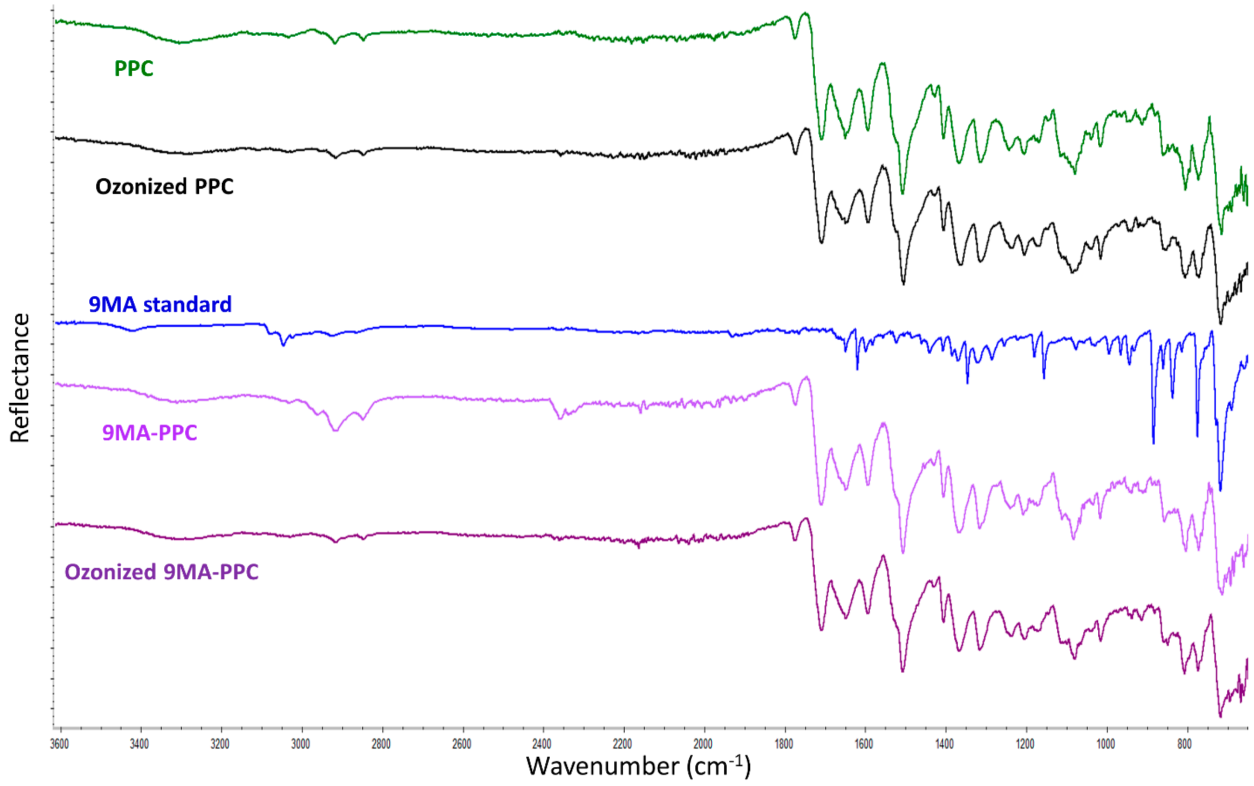

Figure S5. FTIR spectrum of the 9-methylanthracene (9MA) standard and FTIR spectra of PPC and 9MA-impregnated PPC, before and after the ozone treatment for 60 min. The FTIR spectra are vertically offset for clarity.

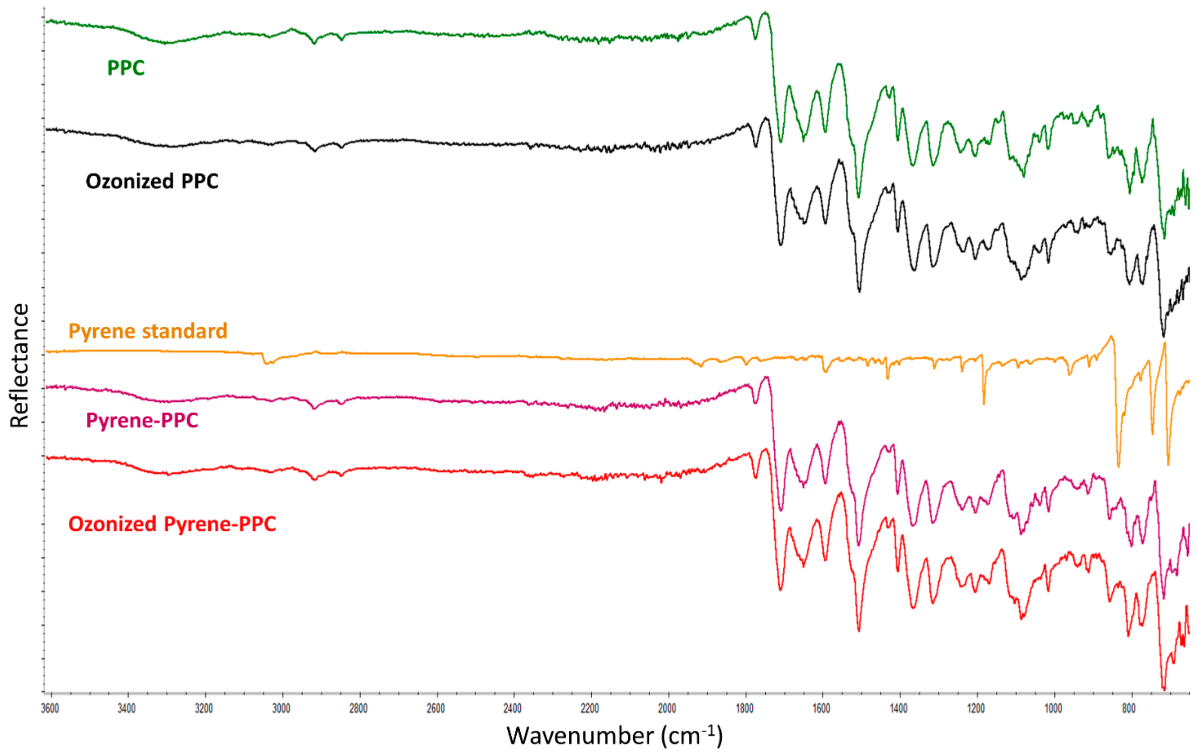

Figure S6. – FTIR spectrum of the pyrene standard and FTIR spectra of PPC and pyrene-impregnated PPC, before and after the ozone treatment for 60 min. The FTIR spectra are vertically offset for clarity.

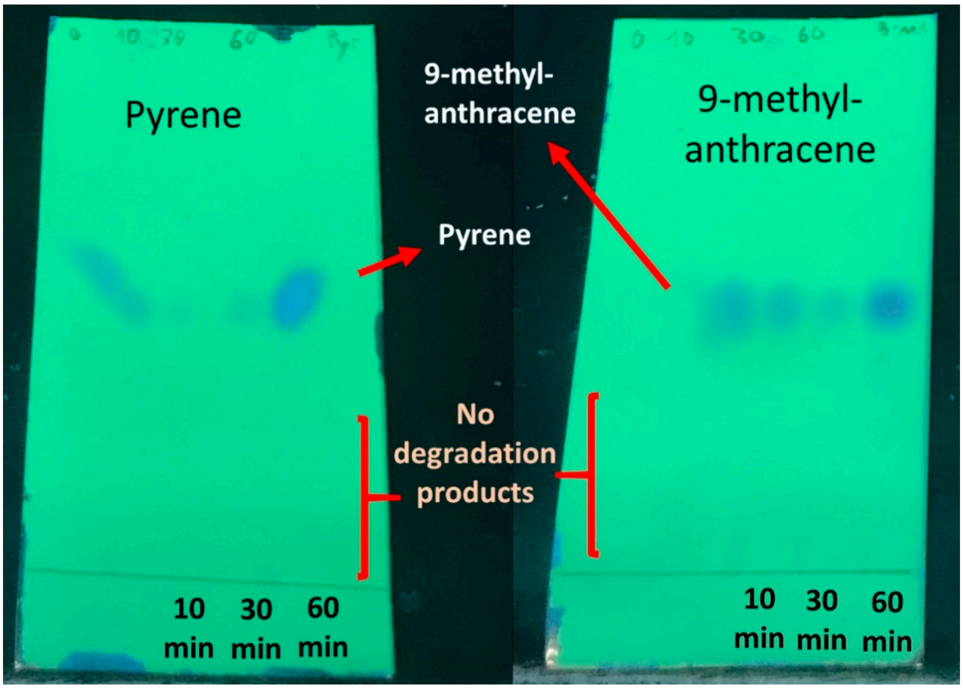

Figure S7. TLC chromatograms of the pyrene (left) and 9-methylantracene (right) solutions extracted from the PPC fragments treated with ozone in the heterogeneous experiments for 10, 30, and 60 min. The images were acquired under UV radiation (254 nm).

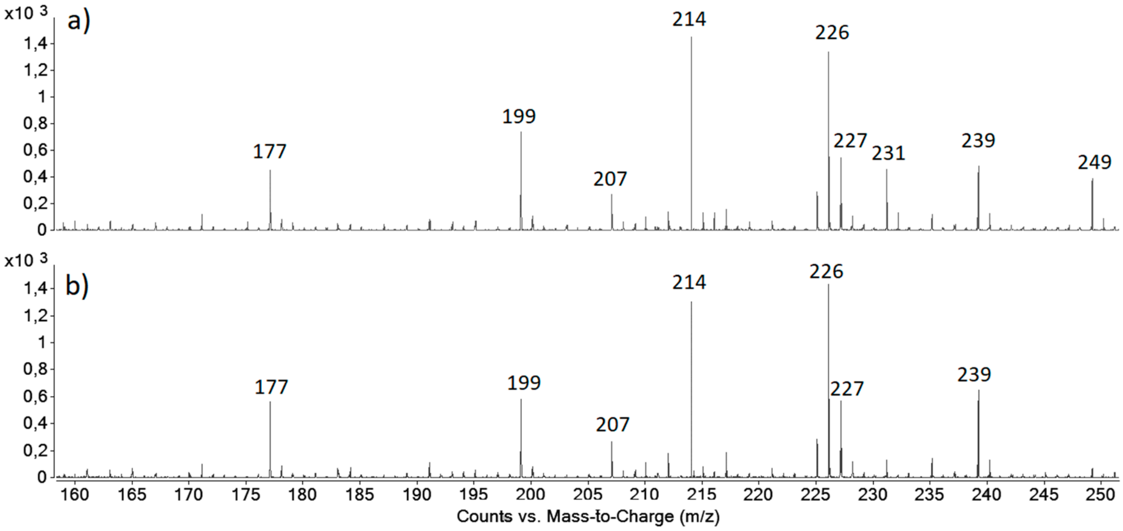

Figure S8. MS spectra of the solution extracted from the (a) unused firefighters' personal protective clothes (PPC) fragment, and (b) unused PPC submitted to ozonolysis for 60 min.
